# High diagnostic rate of trio exome sequencing in consanguineous families with neurogenetic diseases

**DOI:** 10.1093/brain/awab395

**Published:** 2021-11-17

**Authors:** Semra Hiz Kurul, Yavuz Oktay, Ana Töpf, Nóra Zs Szabó, Serdal Güngör, Ahmet Yaramis, Ece Sonmezler, Leslie Matalonga, Uluc Yis, Katherine Schon, Ida Paramonov, İpek Polat Kalafatcilar, Fei Gao, Aliz Rieger, Nur Arslan, Elmasnur Yilmaz, Burcu Ekinci, Pinar Pulat Edem, Mahmut Aslan, Bilge Özgör, Angela Lochmüller, Ashwati Nair, Emily O'Heir, Alysia K Lovgren, Broad Center for Mendelian Genomics, Reza Maroofian, Henry Houlden, Kiran Polavarapu, Andreas Roos, Juliane S Müller, Denisa Hathazi, Patrick F Chinnery, Steven Laurie, Sergi Beltran, Hanns Lochmüller, Rita Horvath

**Affiliations:** 1 Izmir Biomedicine and Genome Center, Dokuz Eylul University Health Campus, Izmir 35340, Turkey; 2 Izmir International Biomedicine and Genome Institute, Dokuz Eylul University, Izmir 35340, Turkey; 3 Department of Paediatric Neurology, School of Medicine, Dokuz Eylul University, Izmir 35340, Turkey; 4 Department of Medical Biology, School of Medicine, Dokuz Eylul University, Izmir 35340, Turkey; 5 John Walton Muscular Dystrophy Research Centre, Institute of Translational and Clinical Research, Newcastle University, Newcastle upon Tyne NE1 3BZ, UK; 6 Epilepsy-Neurology Polyclinic of Buda Children's Hospital, New Saint John's Hospital and Northern -Buda United Hospitals, Budapest 1023, Hungary; 7 Department of Paediatric Neurology, Faculty of Medicine, Turgut Ozal Research Center, Inonu University, Malatya 44210, Turkey; 8 Pediatric Neurology Clinic, Diyarbakir 21070, Turkey; 9 CNAG-CRG, Centre for Genomic Regulation, Barcelona Institute of Science and Technology, Barcelona 08003, Spain; 10 Department of Clinical Neurosciences, School of Clinical Medicine, University of Cambridge, Cambridge Biomedical Campus, Cambridge CB2 0PY, UK; 11 Medical Research Council Mitochondrial Biology Unit, University of Cambridge, Cambridge Biomedical Campus, Cambridge CB2 0XY, UK; 12 Rehabilitation Centre for the Physically Handicapped, Budapest 1528, Hungary; 13 Department of Paediatric Nutrition and Metabolism, School of Medicine, Dokuz Eylul University, Izmir 1528, Turkey; 14 GKT School of Medical Education, King's College London, London SE1 1UL, UK; 15 Program in Medical and Population Genetics, Broad Institute of MIT and Harvard, Cambridge, MA SE1 1UL, USA; 16 Neurogenetics Laboratory, National Hospital for Neurology and Neurosurgery, University College London, London WC1N 3BG, UK; 17 Children's Hospital of Eastern Ontario Research Institute, University of Ottawa, Ottawa ON K1H 8L1, Canada; 18 Leibniz-Institut für Analytische Wissenschaften, ISAS e.V., Dortmund 44227, Germany; 19 Department of Pediatric Neurology, University of Duisburg-Essen, Essen 45141, Germany; 20 Department of Clinical Neurosciences, John Van Geest Centre for Brain Repair, School of Clinical Medicine, University of Cambridge, Cambridge CB2 0PY, UK; 21 Department of Neuropediatrics and Muscle Disorders, Medical Center, Faculty of Medicine, University of Freiburg, Freiburg 79106, Germany; 22 Division of Neurology, Department of Medicine, The Ottawa Hospital; and Brain and Mind Research Institute, University of Ottawa, Ottawa ON K1Y 4E9, Canada

**Keywords:** consanguineous families, neurogenetic disease burden, whole exome sequencing, rate of consanguinity

## Abstract

Consanguineous marriages have a prevalence rate of 24% in Turkey. These carry an increased risk of autosomal recessive genetic conditions, leading to severe disability or premature death, with a significant health and economic burden. A definitive molecular diagnosis could not be achieved in these children previously, as infrastructures and access to sophisticated diagnostic options were limited. We studied the cause of neurogenetic disease in 246 children from 190 consanguineous families recruited in three Turkish hospitals between 2016 and 2020. All patients underwent deep phenotyping and trio whole exome sequencing, and data were integrated in advanced international bioinformatics platforms.

We detected causative variants in 119 known disease genes in 72% of families. Due to overlapping phenotypes 52% of the confirmed genetic diagnoses would have been missed on targeted diagnostic gene panels. Likely pathogenic variants in 27 novel genes in 14% of the families increased the diagnostic yield to 86%. Eighty-two per cent of causative variants (141/172) were homozygous, 11 of which were detected in genes previously only associated with autosomal dominant inheritance. Eight families carried two pathogenic variants in different disease genes. *De novo* (9.3%), X-linked recessive (5.2%) and compound heterozygous (3.5%) variants were less frequent compared to non-consanguineous populations.

This cohort provided a unique opportunity to better understand the genetic characteristics of neurogenetic diseases in a consanguineous population. Contrary to what may be expected, causative variants were often not on the longest run of homozygosity and the diagnostic yield was lower in families with the highest degree of consanguinity, due to the high number of homozygous variants in these patients. Pathway analysis highlighted that protein synthesis/degradation defects and metabolic diseases are the most common pathways underlying paediatric neurogenetic disease. In our cohort 164 families (86%) received a diagnosis, enabling prevention of transmission and targeted treatments in 24 patients (10%).

We generated an important body of genomic data with lasting impacts on the health and wellbeing of consanguineous families and economic benefit for the healthcare system in Turkey and elsewhere. We demonstrate that an untargeted next generation sequencing approach is far superior to a more targeted gene panel approach, and can be performed without specialized bioinformatics knowledge by clinicians using established pipelines in populations with high rates of consanguinity.

## Teaser

In a cohort of 190 consanguineous families, Hiz Kurul *et al*. show that trio whole exome sequencing is superior to a targeted gene panel approach for identifying causative variants in children with neurogenetic disease. A diagnostic yield of 86% was achieved, and diagnosis led to targeted treatment in 10% of patients.

## Introduction

Recent studies show that 10.4% of marriages in the world occur between blood relatives.^1^ This type of marriage has been historically accepted in many populations in the Middle East, West Asia and North Africa, as well as among emigrants from these populations now living in North America and Europe. In Turkey consanguineous marriages have a prevalence rate of 24%,^2^ and carry an increased risk of autosomal recessive genetic conditions^2^ affecting the nervous system and muscle, leading to severe disability or premature death^3^ and a significant health and economic burden. Recent reports show that the rate of union between blood relatives has not declined in Turkey and there is a West-East gradient of consanguinity.^4,5^ The incidence of autosomal recessive diseases is high leading to a serious public health problem.^6^ Approximately 3.7% of children in Turkey (>839 000) have a disability involving either hearing (0.3%), vision (0.4%), speech (0.6%), motor strength (0.6%), or mental functions (1.3%). High costs of diagnostic procedures, frequent hospitalizations and efforts for special care result in a considerable economic burden.^7^

The advance of next-generation sequencing now offers the opportunity to better understand the genetic causes, ultimately providing a definitive diagnosis and some effective treatments for many families.^8^ Genomic approaches have made tremendous headway in finding new genes for Mendelian diseases leading to a significant increase in the number of entries in the Online Mendelian Inheritance in Man (OMIM) database from 4550 monogenic rare diseases (3209 unique genes) on 05/09/2016 up to 6773 (4357 unique genes) on 06/12/2020.^8,9^ Consanguineous families aided the discovery of many novel disease genes.

In an international collaborative research project (CONSEQUITUR) with Paediatric Neurology Departments of Dokuz Eylul University (Izmir), Inonü University (Malatya) and the Memorial Hospital (Diyarbakir) we have investigated the molecular cause of neurogenetic disease in 190 consanguineous Turkish families. At Dokuz Eylul University in Izmir 7000 children are seen per year, almost 40% (∼2750 cases) are thought to be affected by genetic conditions, ∼50% of these in consanguineous families. This ratio is similar in the two other participating centres (Malatya and Diyarbakir) where approximately 18 000–20 000 cases are admitted to paediatric neurology in a year. A definitive molecular diagnosis could not be achieved in these children previously, as infrastructures and access to sophisticated diagnostic options (MRI, comprehensive metabolic testing, muscle biopsies, genetic testing) are limited.^4^

Here we present our investigations to identify the molecular cause of the neurogenetic disease in 190 Turkish consanguineous families by systematic deep phenotyping combined with trio whole exome sequencing (WES) and integration of data in advanced international bioinformatics platforms (RD-Connect).

## Materials and methods

### Patient recruitment, deep phenotyping and pre-screening

We recruited consecutively selected patients and family members from consanguineous families who attended paediatric neurology departments between 2016–2020 in Izmir, Diyarbakir and Malatya (Turkey) with childhood onset (<18 years) neurogenetic disorders. Adult-onset diseases were not included. We considered simple and complex phenotypes in the same way. Inclusion was done consecutively, not randomly, among patients admitted during the study period, who remained undiagnosed with standard methods. Informed consent was obtained according to the Declaration of Helsinki from all research participants (index patients with mental capacity, parents, siblings) enabling genetic testing including WES and international controlled data sharing in RD-Connect. Local Research Ethics Committee approved the study in Turkey (REC 302-SBKAEK). Samples were pseudo-anonymized, processed and stored within the Izmir Biomedicine and Genome Center and the MRC Centre for Neuromuscular Diseases Biobank (National Research Ethics Service, Newcastle and North Tyneside 1 Research Ethics Committee: REC reference number 08/H0906/28+5).

Frequent single nucleotide variants, copy number variations (CNVs) and triplet expansions causing neurogenetic disorders in children were pre-screened by appropriate genetic and metabolic testing (Supplementary Table 1) for spinal muscular atrophy (*SMN1* deletion), Duchenne muscular dystrophy (*DMD* deletions), Charcot-Marie-Tooth disease type 1A (*PMP22* duplication), myotonic dystrophy type 1 (DM1), Friedreich’s ataxia (*FRDA* repeat expansion), Fragile X, (*FMRI* repeat expansion) spastic paraplegia type 4 (*SPG4*), common primary mitochondrial diseases caused by mitochondrial (mt)DNA variants (m.3243A>G, m.8344A>G and the single mtDNA deletion), and in some patients comparative genomic hybridization array, along with a list of Turkish founder mutations. A metabolic screen including alpha-fetoprotein, acylcarnitine profiling, amino acid analysis in blood and urine, organic acid analysis in urine, tandem-MS profiling and very long chain fatty acids have been carried out in most participants.

**Figure 1:**
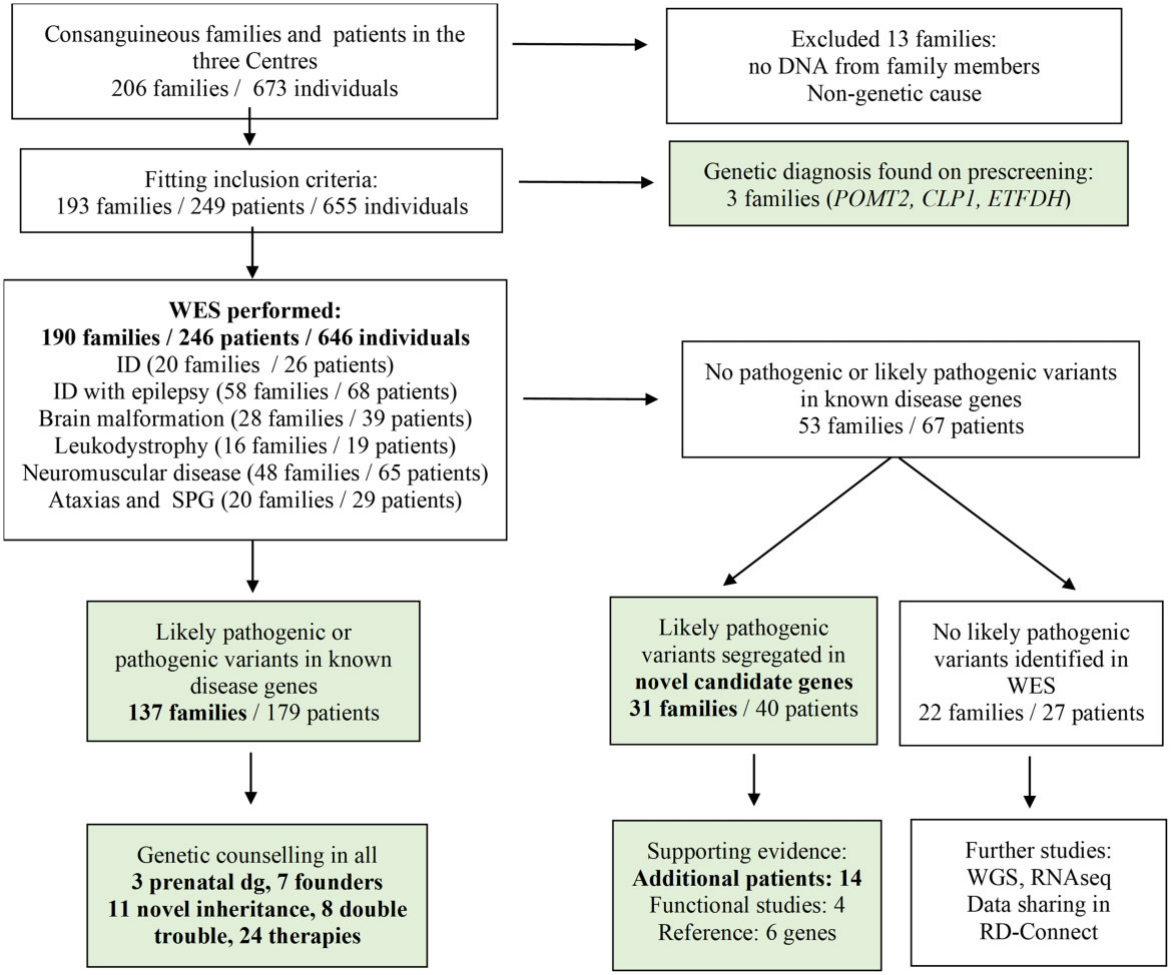
Flow chart of the cohort.

**Figure 2:**
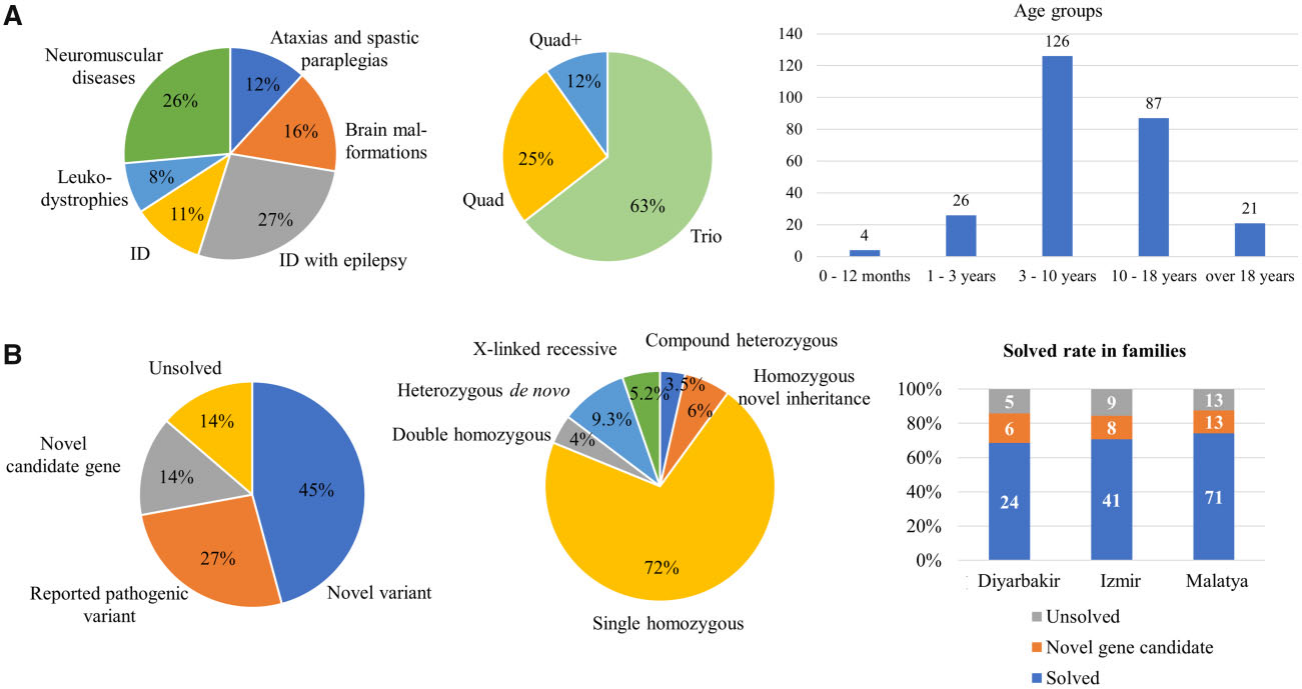
**Description of the cohort, demographic data and main genetic results.** (**A**) Distribution of clinical phenotypes, family structures and age distribution of the patients in our cohort. (**B**) Genetic results, inheritance patterns and distribution of these in the three centres.

The clinical presentations were: (i) intellectual disability (ID); (ii) ID with epilepsy; (iii) brain malformations; (iv) leukodystrophies; (v) ataxia and spastic paraparesis; and (vi) neuromuscular diseases (myopathies, muscular dystrophies, hereditary neuropathies, congenital myasthenic syndromes, mitochondrial disorders). Deep phenotyping and blood sampling of index patients and family members, and in some cases skin or muscle biopsy were performed. The three centres identified 206 families meeting the inclusion criteria. Thirteen families were excluded due to missing consent or low quality of DNA (Fig. 1). In 35 (18%) families two or more affected children were recruited (Fig. 2A). Standard data collection forms were created in order to carry out deep phenotyping of the index patients. For the creation of these forms, terms in the human phenotyping ontology (HPO) database were referenced. Detailed physical examination of each index case was made by paediatric neurologists and phenotypic findings and clinical and laboratory features were recorded. The clinical data of all individuals who underwent WES were uploaded to Phenotips using HPO terms (recently moved to PhenoStore) and are available in the genome phenome analysis platform (GPAP). The results were discussed in regular virtual case rounds with participation of the clinical and genetic team members in Turkey and the UK.

### Whole exome sequencing and bioinformatics analysis

Whole exome sequencing of the index case, parents and affected siblings was performed by the Genomics Platform at the Broad Institute of MIT and Harvard, Cambridge, USA. Libraries were created with an Illumina exome capture (38 Mb target) kit and sequenced with a mean target coverage of >80×. Genomic and phenotypic data were submitted to the RD-Connect GPAP (https://platform.rd-connect.eu), where they can be accessed under a controlled access agreement. Exome sequencing data were processed following GATK3.6 best practices and analysed using the RD-Connect GPAP. Likely pathogenic variants were identified by applying standard filtering for high and moderate impact variants (i.e. nonsense, splice site, frame-shift, in-frame indels and non-synonymous variants), and for minor allele frequency <1% in the genome aggregation database (gnomAD; http://gnomad.broadinstitute.org) and in a cohort of 1182 ethnically-matched Turkish control individuals (TUBITAK MAM-GMBE dataset: http://gmbe.mam.tubitak.gov.tr/en). Shortlisted variants were interrogated for their predicted *in silico* deleteriousness (i.e. combined annotation dependent depletion score >20) and previous known association with human disease, and were classified according to American College of Medical Genetics Guidelines.^10^ We only use the term ‘causative’ when there is a clinical confirmation by the referral centre/clinician.

The typical analysis included the following steps: We run the index individually first and then add the parents in for segregation. This analysis identifies a large number of variants. Indeed, in our cohort, the mean of the number of rare (minor allele frequency < 0.01) variants with a predicted high or moderate impact at the protein level was of 99.01 variants per patient, of which 11.97 were in a homozygous state. Therefore, the ‘index patient only’ analysis often did not reveal the diagnosis. Analysis including parents and siblings in the second step was particularly helpful to resolve the following situations: (i) *de novo* variants; (ii) compound heterozygous variants; (iii) variants homozygous for index and heterozygous for the parents; (iv) variants homozygous for both index and for the parents (exclude); (v) variants homozygous for index and unaffected sibling (exclude); and (vi) variants homozygous for index and not homozygous for affected sibling (exclude). Likely pathogenic variants were segregated in unaffected siblings by Sanger sequencing.

### Copy number variation analysis

Copy number variants were identified from aligned Binary Alignment Map files using ExomeDepth. Events found in any region seen to be CNVs in more than 12 individuals across the cohort were filtered out as being common. Due to the consanguineous nature of the cohort, the search for pathogenic variants focused only on potential homozygous deletions with a reported Bayes Factor greater than 15, and an observed to expected reads ratio less than 0.10, as reported by ExomeDepth. All candidate homozygous CNVs were manually inspected in the integrative genomics viewer in comparison to other available family members to confirm segregation and confirmed to be absent in a homozygous state in the gnomAD-structural variant dataset. We prioritized the identification of homozygous CNVs in genes known to cause rare neuromuscular and neurogenetic diseases as defined by three relevant European Reference Networks (https://ec.europa.eu/health/ern_en): EURO-NMD, ERN-RND and ERN-ITHACA. Then we also searched for homozygous CNVs in the whole exome to discover novel disease genes.

### Mitochondrial DNA analysis

Mitochondrial DNA variants were identified using MToolBox version 1.2.^11^ The workflow includes mapping reads to the rCRS mitochondrial reference sequence and realignment to GRCh37/hg19 nuclear genome to discard the nuclear mitochondrial DNA segment. Once the mtDNA is reconstructed, MToolBox performs variant calling, quantifies heteroplasmy and assigns haplogroups. Low-level heteroplasmy calls (below 10%) were observed in 25% of samples with higher mtDNA coverage (>1000). Variants were further annotated with the latest release of the MITOMAP database (https://www.mitomap.org/MITOMAP). Possible disease-associated variants were identified as those with the status ‘Confirmed’ or ‘Reported’ in MITOMAP.

### Degree of consanguinity and kinship analysis

Runs of homozygosity (ROHs) were identified using the whole genome association analysis toolset PLINK version 1.90^12^ applying the optimized parameters defined in Kancheva *et al*.^13^ This method is designed for WES data and assumes intronic and intergenic regions to be homozygous when surrounded by two detected homozygous coding regions. PLINK was run for each sample to identify all ROHs with a minimum length of 1 Mb. Consanguinity ranges were inferred according to Matalonga *et al*^14^ and samples classified as consanguineous (total ROH size > 123 Mb), probably consanguineous (79 Mb < total ROH size > 123MB), uncertain consanguinity (22 Mb < total ROH size > 79 Mb) and non-consanguineous (22 Mb < total ROH size). Kinship analysis was performed using the relatedness2 option from vcftools. Plots were generated using RStudio version 1.0.143 (RStudio, Boston, MA) and statistical analysis was performed using ANOVA-test and *t*-test (two-sample assuming equal variances).

### Pathway analysis

We have performed enrichment analysis on our gene set using gene ontology (GO) enrichment analysis, which is available in Uniprot (https://www.uniprot.org/). Gene identifiers were converted into Uniprot identifiers and imported into UniProt. Genes mapped to proteins were then classified based on their GO Biological Process and then manually grouped into major classes such as protein production and degradation or metabolism. We have annotated 114 proteins and their corresponding genes from a total of 120 known disease genes.

### Data availability

The exome sequencing data are made available for international controlled data sharing via the European genome-phenome archive and RD-Connect (Ucam-horvath dataset). Accession numbers and codes for the genetic data in RD-Connect are available for RD-Connect partners and available on request for non-partners.

## Results

### Demographics, deep phenotyping and pre-screening

The three paediatric centres recruited 249 patients, their parents and healthy siblings (655 individuals) (Fig. 1). All patients had a childhood-onset condition, the age at recruitment was 0–21 years (median age: 9 years 8 months), >80% between 3–18 years (Fig. 2A). The parental age at the affected child’s birth was 26.31 years for mothers and 29.73 years for fathers, and similarly low for *de novo* cases (mothers: 25.1 years, fathers: 28.4 years). There were 134 male and 112 female patients. Pre-screening revealed the diagnosis in only three families (*POMT2*, *CLP1*, *ETFDH*).

We performed WES in 646 individuals from 190 consanguineous families: 246 patients (including 56 affected siblings), their parents (380) and healthy (20) siblings with ID and epilepsy (68 patients/58 families), pure ID (26 patients/20 families), neuromuscular disease (65 patients/48 families), brain malformations (39 patients/28 families), leukodystrophies (19 patients/16 families), ataxia and spastic paraparesis (29 patients/20 families) (Fig. 2A). Some families had symptoms for more than one disease category.

### Variants in known disease genes

Whole exome sequencing identified 141 likely causative variants in 119 known disease genes in 137 families (72% of our cohort), classified by American College of Medical Genetics criteria^15^ and clinical confirmation by the referral centre/clinician (Supplementary Table 2). Thirty-seven out of 141 variants were identified by the referring clinicians directly, as they were flagged as clearly pathogenic variants in known disease genes in ClinVar. The large share (100/141 variants) was identified at multidisciplinary team meetings including the Turkish clinicians and clinical scientists from the UK and Canada with experience in rare disease research. At these meetings the predicted pathogenic effect was assessed in the light of the clinical presentation to decide whether these are causative for the disease. The remaining four variants (CNVs) were identified by the CNAG bioinformatics team.

Likely pathogenic variants had been previously reported in ClinVar and/or relevant publications or were novel, rare or absent in the control populations and had a high deleterious prediction score (combined annotation dependent depletion score >20) in a gene known to be associated with the patient’s phenotype.^10,16^ The vast majority of genetic diagnoses were ‘private’ mutations identified in single families only, however variants in 21 genes were identified in more than one family (2× *MED12*, *TTC1*, *CLP1*, *SAMHD1*, *CDKL5*, *SYNGAP1*, *SCN2A*, *FOLR1*, *ETFDH*, *SH3TC2*, *GDAP1*, *GNPTG*, *ALS*, *SACS*, *DHX37*, *RNASET2*, *PCDH12*; 3× *ADSL*, *COLQ*, *ASXL3;* 4× *WWOX*). Only seven homozygous variants were detected in more than one family suggesting a potential founder effect in *ETFDH* (c.1130T>C, p.Leu377Pro), *WWOX* (c.716T>G, p.Leu239Arg), *ADSL* (c.268G>A, p.Ala90Thr), *SAMHD1* (c.490C>T, p.Arg164Ter), *CLP1* (c.419G>A, p.Arg140His), *COLQ* (c.414G>A, p.Trp138Ter) and *TTC1* (c.784T>G, p.Phe262Val). Deep phenotyping of the patients with HPO terms enabled the use of the Exomiser program to match genotype to phenotype by an automated method.^13^ We estimate that Exomiser analysis was contributory in 1/3 of the novel genes in our cohort.

**Figure 3:**
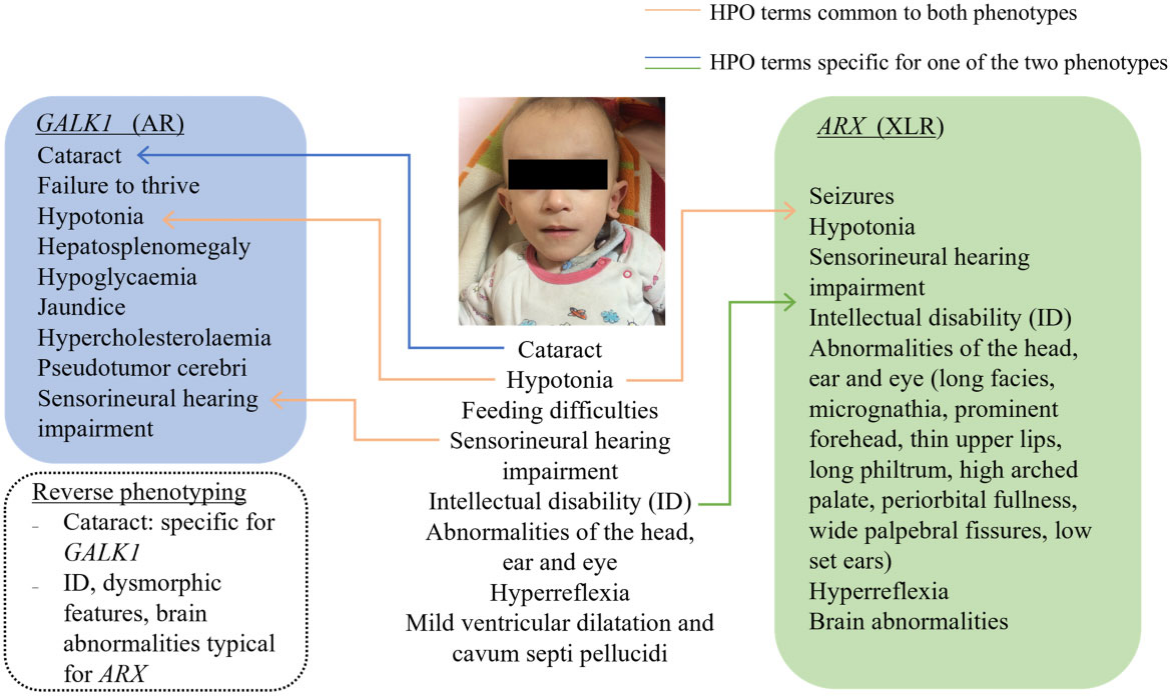
**HPO-term phenotyping of patients with ‘double trouble’ genetic diagnosis.** In this patient the cataract is compatible with variants in *GALK1*. The severe psychomotor delay, dysmorphic features, hyperreflexia and brain abnormalities are explained by the *ARX* variant.

The detection rate of likely causative variants in known disease genes was highest in ataxias and spastic paraparesis (90%), followed by neuromuscular diseases (77%), leukodystrophies (75%), brain malformations (75%) and ID with epilepsy (66%), and lowest in pure ID (55%; Supplementary Tables 3 and 4). While some phenotypes observed in the patients expanded the clinical spectrum of some known disease genes,^17^ in other families the phenotype associated with the suspected disease-causing gene did not match the patient’s clinical presentation, and reassessment of the phenotype (i.e. reverse phenotyping) was required (Fig. 3 and Supplementary Fig. 1). It is important to note that 72 out of 137 families (52%) with a genetic diagnosis in a known disease gene would have been missed by frequently used gene panel analysis (Supplementary Table 5).

### Variants in novel candidate genes

We identified 28 likely pathogenic variants in 36 patients (27 families) in 27 novel genes not yet associated with human disease in OMIM (before 31/12/2020; Table 1). Almost half of the novel candidate genes were detected in ID with epilepsy (13/27; 48%). Two of these genes (*DHX37*, *UFSP2*) were identified in more unrelated families in this cohort with matching phenotype, supporting the pathogenicity. International matchmaking utilizing the RD-Connect GPAP database identified additional families with matching phenotypes for 14 genes, supporting that these are indeed likely to be causative for the disease, and functional studies are ongoing. Some evidence supports the pathogenic role of two of the remaining 13 novel candidate genes (Table 1). Confirmation of the pathogenic nature of the variants in these genes will need to be reviewed in the future.

**Table 1:** Novel candidate genes identified in this study

Gene	Zygosity	Variant	Disease group
*KATNAL2* ^a^	Homozygous	c.1174T>C, p.Ser392Pro	ID with epilepsy
*GFRA1*	Homozygous	c.611C>T, p.Pro204Leu	ID with epilepsy
*CCDC82* ^a^	Homozygous	c.1036delG, p.Ala346LeufsTer3	ID with epilepsy
*XAB2*	Homozygous	c.2047C>T, p.Arg683Cys	ID with epilepsy
*MAN1A2* ^a^	Homozygous	c.553A>T, p.Lys185Ter	ID with epilepsy
*EEF1D* ^18^	Homozygous	c.947G>A, p.Trp316Ter	ID with epilepsy
*UBAP2* ^a^	Homozygous	c.970A>G, p.Ile324Val	ID with epilepsy
*TFAP2E*	Homozygous	c.671G>A, p.Arg224Gln	ID with epilepsy
*KNDC1* ^a,19^	Homozygous	c.3560T>G, p.Leu1187Trp	ID with epilepsy
*KRBOX4* ^a^	Hemizygous, XLR	c.142G>T, p.Gly48Trp	ID with epilepsy
*DHX37* ^a,20^	Homozygous	c.1105G>A, p.Val369Met	ID with epilepsy
*DHX37* ^a,20^	Homozygous	c.661G>C, p.Ala221Pro	Brain malformation
*WDR91* ^a,21^	Homozygous	c.1395+1G>A	Brain malformation
*SPP1*	Homozygous	c.120C>T(p.=) p.Glu105Ter	Brain malformation
*PTPMT1* ^a^	Homozygous	c.255G>T, p.Gln85His	Brain malformation
*NAA60* ^a^	Homozygous	c.130C>T; p.Arg44Cys	Brain malformation
*CCDC28B*	Homozygous	c.685C>T, p.Pro229Ser	CMT
*ARHGAP19* ^a^	Homozygous	c.451C>A, p.Glu151Lys	CMT
*FBXO34*	Homozygous	c.482A>G, p.Lys161Arg	Myopathy
*NPAP1* ^a^	Homozygous	c.3407C>G, p.Ser1136*	Myopathy
*DDB1*	Homozygous	c.2566+4A>G	Leukodsytrophy
*SPATA5L1* ^a^	Homozygous	c.85T>G; p.Cys29Gly	Leukodsytrophy
*USP38*	Double homozygous	c.2257C>G, p.Gln753Glu c.2489A>T, p.Asp830Val	ID
*HIST1H4C* ^17^	Heterozygous *De novo*	c.275A>G, p.Lys92Arg	ID
*UFSP2* ^a,22^	Homozygous	c.344T>A; p.Val115Glu	Brain malformation
*UFSP2* ^a,22^	Homozygous	c.344T>A; p.Val115Glu	ID with epilepsy
*UFSP2* ^a,22^	Homozygous	c.344T>A; p.Val115Glu	Ataxia
*UFSP2* ^a,22^	Homozygous	c.542delT	Ataxia
*ZNF92* (*CNV*)	Homozygous	ch7:64838860–65113330	Leukodystrophy
*ACSM5* (*CNV*)	Homozygous	ch16:20451071–20451801	ID with epilepsy
*AGBL3*	Homozygous	ch7:134728726–134735844	Brain malformation
Large deletion *K3/CDC37L1/CDC37L1/DT/* *PLPP6/SPATA6L*	Homozygous	ch9:4604129–4722677	ID with epilepsy

Novel candidate genes in 40 patients from 31 families in this study.

a Variant in this gene has been detected in additional patients with similar phenotype or supporting references.

### Copy number variant analysis

We prioritized the identification of homozygous CNVs in genes known to cause rare neuromuscular and neurogenetic diseases as defined by three relevant European Reference Networks (https://ec.europa.eu/health/ern_en): EURO-NMD, ERN-RND and ERN-ITHACA. A homozygous deletion of exon 2 of the *MFSD8* gene is associated with neuronal ceroid lipofuscinosis type 7 and a homozygous deletion of 16 exons in *PARK7* was detected in a child with pathogenic *SACS* mutations (Supplementary Table 2). Homozygous deletions in either *CRB1* or *PLA2G6* were detected in two siblings from a single family, where parents are heterozygous for both deletions. In addition, we identified homozygous deletions affecting novel candidate genes in five further patients from four families (*AGBL3*, *ZNF92*, *ACSM5*, *K3*/*CDC37L1*/*CDC37L1*/*DT/PLPP6*/*SPATA6L*). We did not study heterozygous CNVs in our cohort.

### Mitochondrial DNA analysis

The analysis of mtDNA detected four heteroplasmic, but no homoplasmic, variants previously associated with mitochondrial disease. The common m.3243A>G variant with 4.2% heteroplasmy rate was present in one of two affected siblings carrying a homozygous *COL18A1* variant, but in none of his maternal relatives, making it unlikely to contribute to the phenotype. A m.10197G>A variant with 4.6% heteroplasmy was detected in a patient with confirmed Vici syndrome due to a homozygous splice variant in *EPG5*, which was not present in any of the maternal family members. The m.4308G>A variant was detected with 1% heteroplasmy in the unaffected mother of a child with a homozygous mutation in a novel candidate gene (*MAN1A2*), and the 89% heteroplasmic m.14484T>C known Leber’s hereditary optic neuropathy variant was present in the unaffected father of a child with a homozygous variant in a novel candidate gene (*KNDC1*). This variant may increase the risk to the father of developing Leber’s hereditary optic neuropathy but does not contribute to the disease in the child.

### Inheritance patterns

The majority (82%) of likely causative variants (141/172) were homozygous, including homozygous variants in 11 genes previously only associated with autosomal dominant disease (Table 2), where heterozygous parents and siblings were healthy. The detection rate of *de novo* (*n* = 16, 9.3%, including five X-linked dominant), X-linked recessive (*n* = 9, 5.2%) and compound heterozygous (*n* = 6, 3.5%) variants across the whole cohort was lower compared to 30–50% in non-consanguineous populations.^23,24^ Most *de novo* (*n* = 11, 17%) and X-linked recessive (*n* = 6, 9%) variants were detected in patients with ID with or without epilepsy, but still lower than expected in ID, while *de novo* variants were hugely underrepresented in the other disease groups. We detected seven families with two homozygous pathogenic variants in the index patient (double trouble), while in another family two different disease genes caused the different phenotype of two affected siblings. Systematic study of the HPO terms facilitated the confirmation of ‘double trouble’ genetic diagnoses (Fig. 3). The double trouble cases were discussed at multidisciplinary team meetings, and our experienced paediatric neurologists from Turkey pointed out, where the phenotype of a patient would be more severe than expected for a particular gene or include additional signs and symptoms that are not described for this gene. Therefore, in some double trouble cases we could not fully confirm the causative role of the second variant, which is a limitation of our study.

**Table 2:** Novel autosomal recessive inheritance pattern in genes previously known with autosomal dominant or *de novo* inheritance

Gene	Zygosity	Variant	Reported phenotype
*COL4A1*	Homozygous	c.3832G>A; p.Gly1278Ser	Microangiopathy and leuko-encephalopathy, pontine, AD
*POLR1A*	Homozygous	c.4498C>T; p.Arg1500Cys	Acrofacial dysostosis, Cincinnati type, AD
*TLK2*	Homozygous	c.163A>G; p.Lys55Glu	Mental retardation, AD, 57
*CACNA1S*	Homozygous	c.2366G>A; p.Arg789His	Hypokalemic periodic paralysis, type 1, AD
*ASH1L*	Homozygous	c.7756G>A; p.Gly2591Ser	Mental retardation, AD, 52
*PRKCG*	Homozygous	c.1769T>C; p.Leu590Pro	Spinocerebellar ataxia 14, AD
*KMT2C*	Homozygous	c.13174C T; p.Pro4392Ser	Kleefstra sy/Lissencephaly/Epilepsy temp lobe, AD
*HMBS*	Homozygous	c.500G>A; p.Arg167Gln	Porphyria, acute intermittent, AD
*CACNB4*	Homozygous	c.8C>T; p.Ser3Phe	Episodic ataxia, type 5, AD Epilepsy juvenile myoclonic, AD
*SCN2A*	Homozygous	c.1976G>A; p.Gly659Asp	Epileptic encephalopathy, early infantile, 11, AD
*TMEM240*	Homozygous	c.47C>A; p.Ser16Ter	Spinocerebellar ataxia 21, AD

AD = autosomal dominant.

### Identity by descent analysis confirmed the rate of consanguinity

**Figure 4:**
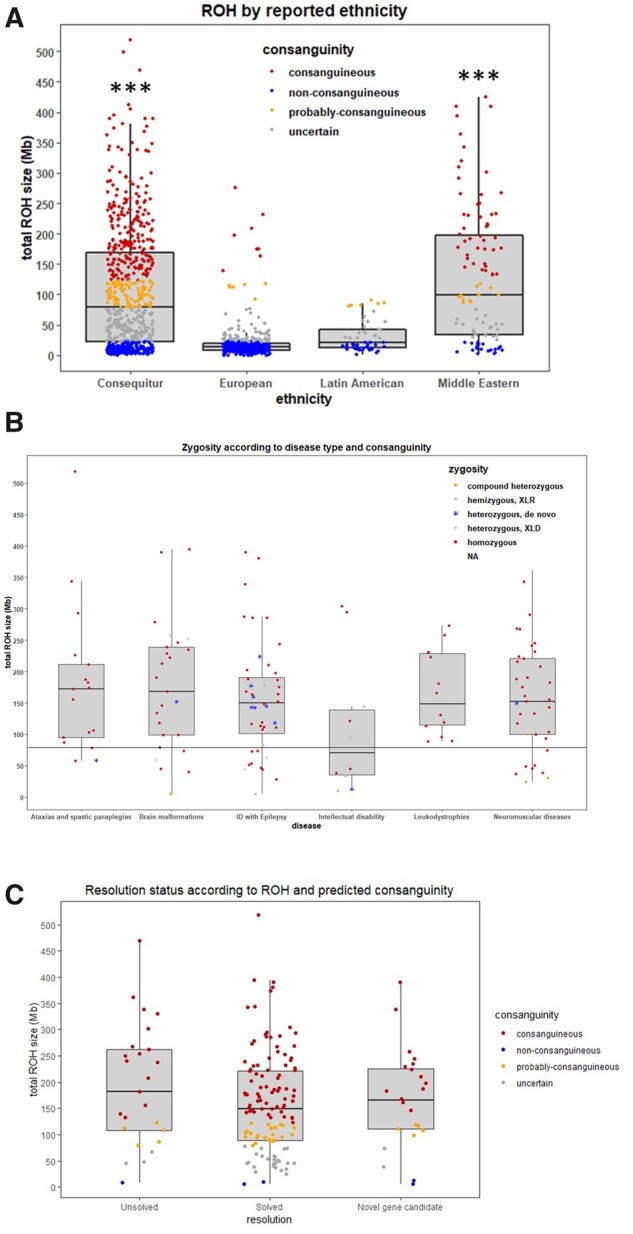
**Consanguinity assessment across the Consequitur cohort.** (**A**) Total ROH size distribution of the whole Consequitur cohort (*n* = 646 individuals including index cases and relatives) compared with other ethnicities from the RD-Connect GPAP. Consanguinity ranges are defined according to Matalonga *et al*.^14^ [consanguineous (total ROH size > 123 Mb), probably consanguineous (79 Mb < total ROH size > 123 Mb), uncertain consanguinity (22Mb < total ROH size > 79 Mb) and non-consanguineous (22 Mb < total ROH size)]. ***P-value < 0.001. (**B**) Total ROH size distribution according to disease type and causative variant zygosity. Black line represents the consanguinity threshold (79 Mb). (**C**) The distribution of the total ROH size according to the resolution status (solved/unsolved) of all index cases.

Identity by descent analysis indicated that in the majority of the 190 families the index cases were highly consanguineous (61%) and probably consanguineous (19%), 17% were possibly consanguineous and 3% did not appear to be consanguineous, although the parents reported consanguinity. Compared to other ethnicities (European and Latin Americans) processed identically in the RD-Connect GPAP,^14^ this cohort showed a significant increase in the total ROH size (Fig. 4A) and were similar to Middle Eastern populations. The median total ROH from index cases were all above the consanguinity threshold defined previously,^14^ except for ID (Fig. 4B). Notably, 11 of the 16 *de novo* pathogenic variants were identified in patients with ID (Fig. 3B). In total 31 non-homozygous pathogenic variants (compound heterozygous, X-linked recessive/dominant or heterozygous *de novo*) were detected (Supplementary Table 2). Of these, 18 were identified in patients with consanguineous genetic backgrounds on the basis of ROH, of which 13 had parents whose kinship coefficient suggested first cousins or closer, and 4 were second cousins. This suggests that 16% (18/115) non-homozygous variants occur also in consanguineous families. Twenty homozygous variants were identified in cases with uncertain consanguinity status and none in predicted non-consanguineous cases (Fig. 4C).

The causative variant was in the longest ROH in 10% of cases which is proportionate to the average contribution (13%) of the longest detected ROH to the total length of homozygous runs (Total SumRoH) across the 229 highly consanguineous individuals in the cohort. We demonstrate that there is no reason *per se* to expect the causative variant to be in the longest ROH. The median of the total ROH was slightly lower for solved cases, suggesting that identifying the causative variant is more difficult in families with higher ROH, due to the presence of several homozygous variants (Fig. 4C). Families with two homozygous pathogenic variants are in the first quartile of consanguinity rates and the two variants were located to different ROHs in all cases.

### Mapping the genetic landscape of paediatric neurogenetic diseases

**Figure 5:**
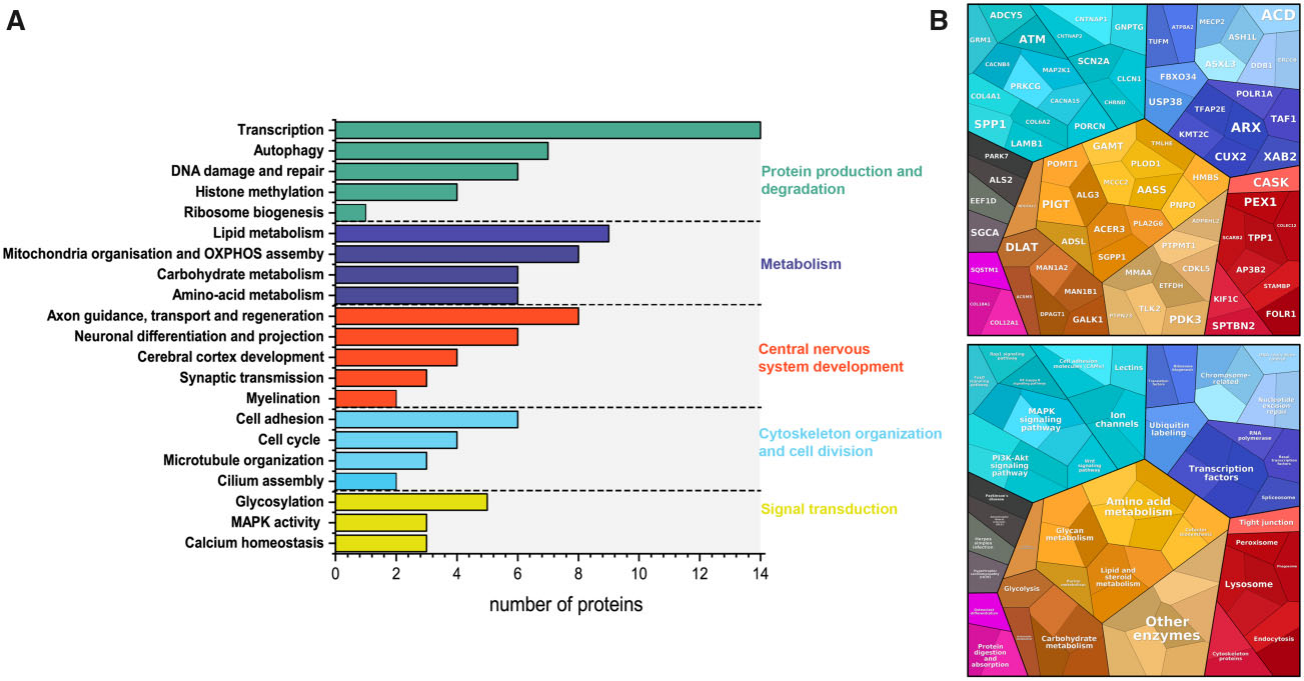
**Molecular pathways affected.** (**A**) GO enrichment analysis of genes and proteins leading to paediatric neurogenetic disease in our cohort. Gene IDs were converted into Uniprot IDs and imported into Uniprot. Genes mapped to proteins were then classified based on their GO biological process and then manually grouped into major classes such as Protein production and degradation or metabolism. (**B**) Proteomap of detected disease genes in our cohort.

We have annotated 114 proteins from a total of 119 disease genes. GO enrichment analysis on our gene set identified five major pathways divided in multiple sub-pathways that are predominantly altered in our patient cohort with the top three being transcription, lipid metabolism and mitochondrial organization and OXPHOS assembly (Fig. 5A and B).

### Treatable human diseases and prevention of disease transmission

We have identified potentially treatable genetic causes in 24 patients (19 families). Rapid communication with the treating clinician in Turkey led to effective treatment in 12 of the 24 patients and made a huge impact for their families (Table 3). The genetic diagnosis enabled genetic counselling, which was given to all 190 families. Most families decided not to have more children after the sick child. Prenatal genetic diagnosis was performed in one family in Izmir and to two families in Malatya.

**Table 3:** Potentially treatable diseases

Gene	Published phenotype	OMIM	Treatment	Clinical outcome
*TPP1 n* = 1	Ceroid lipofuscinosis, neuronal, 2	204 500	Cerliponase alfa	Medication was initiated, will be followed up regularly
*GAMT n* = 1	Cerebral creatine deficiency syndrome 2	612 736	Creatine-monohydrate, L-ornithine, sodium benzoate low protein diet	Seizures stopped, independent walking and understanding improved; two similarly affected cousins were diagnosed and treated immediately, they develop well, have no seizures. Video about the index: https://irdirc.org/yakups-journey-to-hope/
*DLAT n* = 1	Pyruvate dehydro-genase E2 deficiency, Leigh sy -like	245 348	Ketogenic diet	Died before genetic diagnosis
*FOLR1 n* = 5	Neurodegeneration due to cerebral folate transport deficiency	613 068	Folinic acid, vitamin B6	Alertness, perception and vocabulary improved in all 5 patients, unfortunately 3 lost follow up
*PNPO n* = 1	Pyridoxamine 5'-phosphate oxidase deficiency	610 090	Pyridoxine	Started, will be followed up
*GALK1* *n* = 1	Galactokinase deficiency with cataracts	230 200	Dietary lactose restriction	Prevented cataract, no change in psychomotor function
*ETFDH* *n* = 2	multiple acyl-CoA dehydrogenase deficiency MADD	231 680	Riboflavin, coq10	Muscle strength improved in a few weeks with better head control, started independent walking at 3 years of age, after treatment
*COQ4 n* = 1	Coenzyme Q10 deficiency, primary 7	616 276	Coq10	Had severe epilepsy and died soon after diagnosis
*COLQ n* = 4	Myasthenic syndrome, congenital, 5	603 034	Ephedrine, salbutamol	Fatigue improved on salbutamol for 1 year, they avoid mestinon; used to get tired after 10 steps, can now take 50 steps. He can ride his bike and plays football. His father states his recovery is 40%.
*AASS n* = 1	Hyperlysinaemia type 1	238 700	Dietary lysine restriction	Initiated, will be followed up
*MMAA n* = 1	Methylmalonic aciduria, cb1A type	251 100	Vitamin B12	Initiated, will be followed up
*SLC39A8 n* = 1	Congenital disorder of glycosylation, type IIn	616 721	Galactose and manganese	Discussed with the family, scheduled for follow up
*CHRND* *n* = 1	Myasthenic syndrome, congenital, 3	100 720	Pyridostigmine	Improved muscle strength
*HMBS n = 1*	Porphyria, acute intermittent, AD	176 000	Hemin, liver transplantation	Discussed with the family, scheduled for follow up
*CLCN1 n = 2*	Myotonia congenita, autosomal recessive	255 700	Mexiletine, quinine, acetazolamide	Mexiletine not available, others started

## Discussion

Current diagnostic procedures for neurogenetic diseases are cumulatively expensive and time consuming, often do not provide a definite diagnosis, and only rarely lead to effective treatment, resulting in emotional and financial burden for families and society. By exome sequencing of trios and affected siblings we identified the molecular cause of 86% of 190 Turkish consanguineous families. This is much higher than detected in the UK (∼37%),^25^ that also includes immigrant populations (e.g. from Turkey) with consanguinity and high birth rates. In a UK rare disease cohort (100k genome pilot) the diagnostic yields of whole genome sequencing for likely Mendelian monogenic disorders reached 37%, while the highest yield was 40–50%, detected in trios and larger pedigrees with intellectual disability, hearing and eye disorders.^25^ Patients from consanguineous marriages are often seen at specialist clinics, providing a significant burden for the US, UK and European National Health Services. In fact, many of the recent gene discoveries were based on the investigation of consanguineous immigrant families, particularly from Turkey (e.g. *TACO1*, *SDHAF1*, *C19orf12*).^26,27^ There has been immigration into Turkey from neighbouring countries such as Syria with even higher consanguinity rate, lack of sequencing and bioinformatics infrastructure has precluded them from taking full advantage of next generation sequencing.

Our findings improved outcomes for most affected families. A total of 164 families (86%) received a likely genetic diagnosis enabling prevention of transmission and targeted treatment in 24 patients. Thirty-seven variants were listed in ClinVar as pathogenic or likely pathogenic, while 19 were variants of unknown significance, and our data support the pathogenicity. However, the majority of variants have not been reported previously, suggesting private mutations. Only seven variants occurred in more than one family (possible Turkish founder), pointing to the large genetic heterogeneity of childhood-onset neurological diseases. Having patients from three different geographical parts of Turkey may also contribute to this genetic heterogeneity, although the composition of the cohort was similar in the three recruiting centres. We detected likely causative variants in 27 novel candidate genes and identified matching patients in GPAP in 14 of these families.

As expected, homozygous mutations were detected in the majority of families (82%), while causative *de novo* (*n* = 16, 9.3%), X-linked recessive (*n* = 9, 5.2%), compound heterozygous (*n* = 6, 3.5%) and mtDNA variants (*n* = 0) were much less represented than expected for outbred populations. Most *de novo* and X-linked recessive variants were detected in patients with ID (with or without epilepsy), with still lower frequency (17% *de novo*, 9% X-linked recessive) compared to other ID cohorts.^23,24^ This may have been influenced by the younger parental age (median age of mother 25.1 years, father 28.4 years) in our cohort.^28^ Incorporation of exome-based CNV analysis has been recently reported to be a powerful tool in trio-WES, as it increased the clinical diagnosis with 18.92% (14/74) in a cohort of neurodevelopmental disorders in mostly non-consanguineous families.^29^ The overall diagnostic yield of the CNV analysis in our cohort was 4.2%, which is lower than in other cohorts, which can be explained by the fact that our families were all consanguineous, with a higher risk of having autosomal recessive diseases. The prevalence of mtDNA variants may not be influenced by the consanguinity in our cohort, however our cohort size it too small to estimate it exactly. Although we detected 4 potentially pathogenic mtDNA variants in 646 individuals, none of them was causative, which is compatible with the minimum 1 in 5000 prevalence of mitochondrial DNA related disease.^30^

We identified novel inheritance patterns by detecting homozygous, recessive variants in 11 genes previously only associated with autosomal dominant disease (Table 1). We detected seven patients with two homozygous causative variants in different disease genes (double trouble) and two different recessive diseases in another family (4.2%). This number may be higher if we consider also heterozygous CNVs and all clinical features, however here we focused on neurogenetic manifestations. Second pathogenic or likely pathogenic variants leading to recessive disorders were previously detected in ∼10% of consanguineous couples, as compared to <1% in non-consanguineous couples,^31^ which should be considered when offering prenatal or preimplantation genetic testing. Genetic counselling was performed in all 190 families in this study and most families decided not to have more children; prenatal genetic diagnosis was only performed in three families.

Identity by descent analysis confirmed the high rate of consanguinity in our cohort, except for the six compound heterozygous families. The degree of consanguinity in the *de novo* and X-linked families was similar to homozygous and double homozygous families. Our data provide evidence that in line with expectations, there is no reason *per se* to expect the causative variant to be in the longest ROH. The identification of causative variants was not easier in families with the highest rate of consanguinity. The consanguinity median (total ROH size) is slightly lower for solved than unsolved cases (not significant), reflecting the difficulty to identify the causative variants in families with several homozygous variants.

Interestingly, the >100 molecular mechanisms linked to the 119 disease genes in our cohort are part of a handful of large clusters of pathways, such as transcription, protein synthesis and metabolism. Identifying common molecular targets within these pathways will facilitate the development of treatments in children with these disabling neurological diseases, however, most of them may require distinct treatment approaches, as we have learned in the last decade.

Our study provides useful information on genetic variants in the Turkish population, which will further improve the diagnostic yield and facilitate genomic research in Turkey. Families without a genetic diagnosis will be further analysed by whole genome sequencing including new technologies (long read, etc) and transcriptomic studies, which will likely lead to detection of additional causative variants in these families (repeats, CNVs, intronic variants leading to mis-splicing etc.),^15^ and occasionally patients may have a non-genetic aetiology.

In addition, a small but increasing number of genetically defined neurological diseases (congenital myasthenic syndromes, metabolic defects) are treatable if an accurate molecular diagnosis is obtained early in the disease course. In our study, 24 patients from 19 families (10%) have a potentially treatable cause, and 12 of these patients received treatment based on the new genetic diagnosis.

Pre-screening is expensive, time-consuming and had a very low detection rate in our cohort (1.5%). Next generation gene panels, which are most commonly used in diagnostic testing would have missed the causative variant in 72 out of 137 families (52%) (Supplementary Table 5), clearly demonstrating that WES has much higher diagnostic yield (86%), it is more cost-effective, easy to handle (needs only banked genomic DNA) and enables a rapid detection of a huge variety of human diseases. This is supported by other studies. In an Australian study the diagnostic yield of singleton-WES with simulated application of commercial gene panels in children suspected of having a genetically heterogeneous condition was made in genes not included in at least one-of-three commercial panels in 42% of cases.^32^ In this study 23% of WES-diagnosed children would not have been diagnosed and in 26% of cases the least costly panel option would have been more expensive than WES. The broader coverage of WES makes it a superior alternative to gene panel testing at similar financial cost for children with suspected complex monogenic phenotypes.^32^ A targeted panel approach cannot be used for new gene discovery, as such panels consist of already identified genes, and *de novo* mutations can also only be identified by trio analysis.

Retrospectively, it is difficult to estimate how many cases would have been solved by a singleton versus trio WES approach in our cohort. If we compare the detection yield in our consanguineous cohort with other non-consanguineous cohorts^25^ we can estimate that the detection rate is about two times higher in consanguineous families.

We set up a proof-of-concept that referring clinicians from Turkey were able to analyse their patients’ next generation sequencing data through a standardized bioinformatics pipeline and a remote, federated system (Supplementary Fig. 2). A similar setting would have major impact in identifying the burden of neurogenetic diseases in other countries with high rate of consanguinity, but also in certain ethnic communities in the UK and elsewhere.

## Supplementary Material

awab395_Supplementary_Data

## Abbreviations

CNV: copy number variation
GPAP: genome phenome analysis platform
HPO: human phenotyping ontology
ID: intellectual disability
ROH: run of homozygosity
