# Supplementary material for: High diagnostic rate of trio exome sequencing in consanguineous families with neurogenetic diseases
Source: Brain. 2021 Nov 17;145(4):1507–18. doi: 10.1093/brain/awab395 (PMC9128813; doi:10.1093/brain/awab395)
Supplement: awab395_Supplementary_Data [file awab395_supplementary_data.zip › brain-2021-01013-File007.pdf]

SUPPLEMENTARY INFORMATION

High diagnostic rate of trio exome sequencing in consanguineous families with neurogenetic diseases

Semra Hiz Kurul, MD[1,2,3]*, Yavuz Oktay, PhD[1,4]*, Ana Töpf, PhD[5]*, Nóra Szabó, MD[6,]*, Serdal Güngör, MD[7], Ahmet Yaramis, MD[8], Ece Sonmezler[2], Leslie Matalonga, PhD[9], Uluc Yis, MD[3], Katherine Schon, MD[10,11], Ida Paramonov, PhD[9], İpek Polat Kalafatcilar, MD[2,3], Fei Gao, PhD[10,11], Aliz Rieger, MD[12], Nur Arslan, MD[1,13], Elmasnur Yilmaz[2], Burcu Ekinci[2], Pinar Pulat Edem, MD[3], Mahmut Aslan, MD[7], Bilge Özgör, MD[7], Angela Lochmüller[14], Ashwati Nair[14], Emily O'Heir, PhD[15], Alysia K. Lovgren, PhD[15], Broad Center for Mendelian Genomics, Reza Maroofian, PhD[16], Henry Houlden, MD, PhD[16], Kiran Polavarapu, MD[17], Andreas Roos, PhD[17,18,21], Juliane S. Müller, PhD[10,20], Denisa Hathazi, PhD[10,20], Patrick F. Chinnery, FRCP FMedSci[10,11], Steven Laurie, PhD[9], Sergi Beltran, PhD[9], Hanns Lochmüller, MD, PhD[9,17,10,21]*, Rita Horvath, MD, PhD[10,20]*

Supplementary Figure 1. Reverse phenotyping.

The identification of the "double trouble" cases has been done by lot of caution and we only called patients "double trouble" if they carry 2 causative variants with highest level of evidence of pathogenicity, such as homozygous nonsense variants or reported pathogenic variants in known disease genes. In some cases the HPO terms may not fully match and can be fuzzy.

A. We identified a homozygous *SNX14* variant, which explained the patient`s phenotype, however at the time of recruitment the patient had no ataxia or cerebellar atrophy on MRI. These findings became apparent as the child grew older and are compatible with *SNX14*.. We also detected an additional hemizygous deletion of 5 exons in *TMLHE*. A hemizygous exon 2 deletion of *TMLHE* has been detected previously in a boy[1] which was suggested to be the cause of developmental delay and autism, however another paper from the same authors reported that this genetic variant may be also present in controls and it is a susceptibility locus for autism[2]. Therefore it is possible that the hemizygous *TMLHE* deletion of 5 exons may not lead to autism in our patient, however it is possible that it contributes to the delayed psychomotor development and absent speech (also symptoms of *SNX14*)

B. Some common HPO terms are fitting the phenotypes of both genes, while pachygyria and brain calcifications are key features for *OCLN*. The *OCLN* variant explains the phenotype, however we detected an additional homozygous reported ClinVar pathogenic *MCCC2* variant, which has a low penetrance. This variant has been reported even within the same families with different phenotypes, and some homozygous carriers are asymptomatic, suggesting reduced penetrance[3]. Although this variant does not result in symptoms and metabolic changes at the moment in our patient, it may lead to metabolic decompensation and clinical manifestation later in life.

A

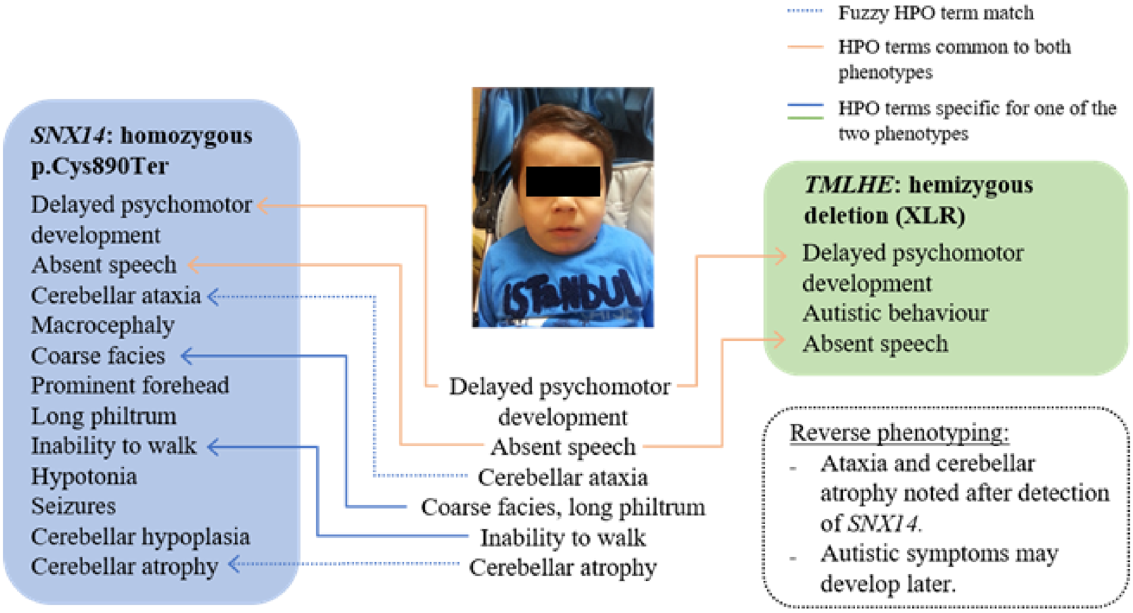

Fuzzy HPO term match
HPO terms common to both phenotypes
HPO terms specific for one of the two phenotypes
SNX14: homozygous p.Cys890Ter
Delayed psychomotor development
Absent speech
Cerebellar ataxia
Macrocephaly
Coarse facies
Prominent forehead
Long philtrum
Inability to walk
Hypotonia
Seizures
Cerebellar hypoplasia
Cerebellar atrophy
Delayed psychomotor development
Absent speech
Cerebellar ataxia
Coarse facies, long philtrum
Inability to walk
Cerebellar atrophy
TMLHE: hemizygous deletion (XLR)
Delayed psychomotor development
Autistic behaviour
Absent speech
Reverse phenotyping:
- Ataxia and cerebellar atrophy noted after detection of *SNX14*.
- Autistic symptoms may develop later.

B

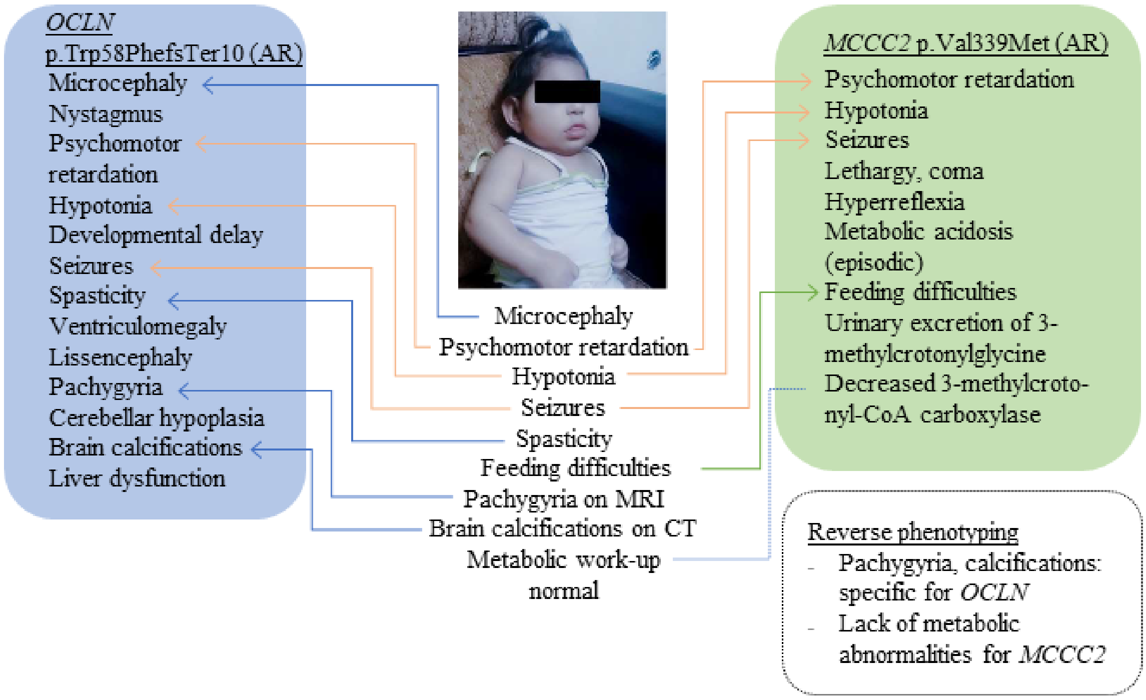

OCLN p.Trp58PhefsTer10 (AR)
Microcephaly
Nystagmus
Psychomotor retardation
Hypotonia
Developmental delay
Seizures
Spasticity
Ventriculomegaly
Lissencephaly
Pachygyria
Cerebellar hypoplasia
Brain calcifications
Liver dysfunction
Microcephaly
Psychomotor retardation
Hypotonia
Seizures
Spasticity
Feeding difficulties
Pachygyria on MRI
Brain calcifications on CT
Metabolic work-up normal
MCCC2 p.Val339Met (AR)
Psychomotor retardation
Hypotonia
Seizures
Lethargy, coma
Hyperreflexia
Metabolic acidosis (episodic)
Feeding difficulties
Urinary excretion of 3-methylcrotonylglycine
Decreased 3-methylcrotonyl-CoA carboxylase
Reverse phenotyping
- Pachygyria, calcifications: specific for *OCLN*
- Lack of metabolic abnormalities for *MCCC2*

Supplementary Figure 2. Data flow in the GPAP database.

The RD-Connect GPAP (https://platform.rd-connect.eu/) is a recognised IRDiRC resource that provides a sophisticated and user-friendly online analysis system for rare disease gene discovery and diagnosis. The GPAP enables integrated analysis of phenotypic and genomic data and tiered data discoverability through GA4GH beacon and MatchMaker Exchange. The GPAP has currently more than 18,000 geno-pheno datasets from 250 groups.

1. Clinical specialist registers to the RD-Connect GPAP (DAC validation required)
2. Raw genomic data is sent to the system (including experiments metadata and FASTQ/BAM files)
3. Standardised phenotypic information is entered in the system (HPO, ORDO, OMIM) through a dedicated phenotypic module (PhenoStore)
4. Genomic data is processed through a standardised pipeline, integrated with the phenotypic information and made accessible to users through a powerful and user-friendly interface
5. Clinical specialists access the RD-Connect GPAP to analyse their cases.
6. Clinical specialists return molecular results from the analysis performed to patients. In some cases, treatment options are also available.

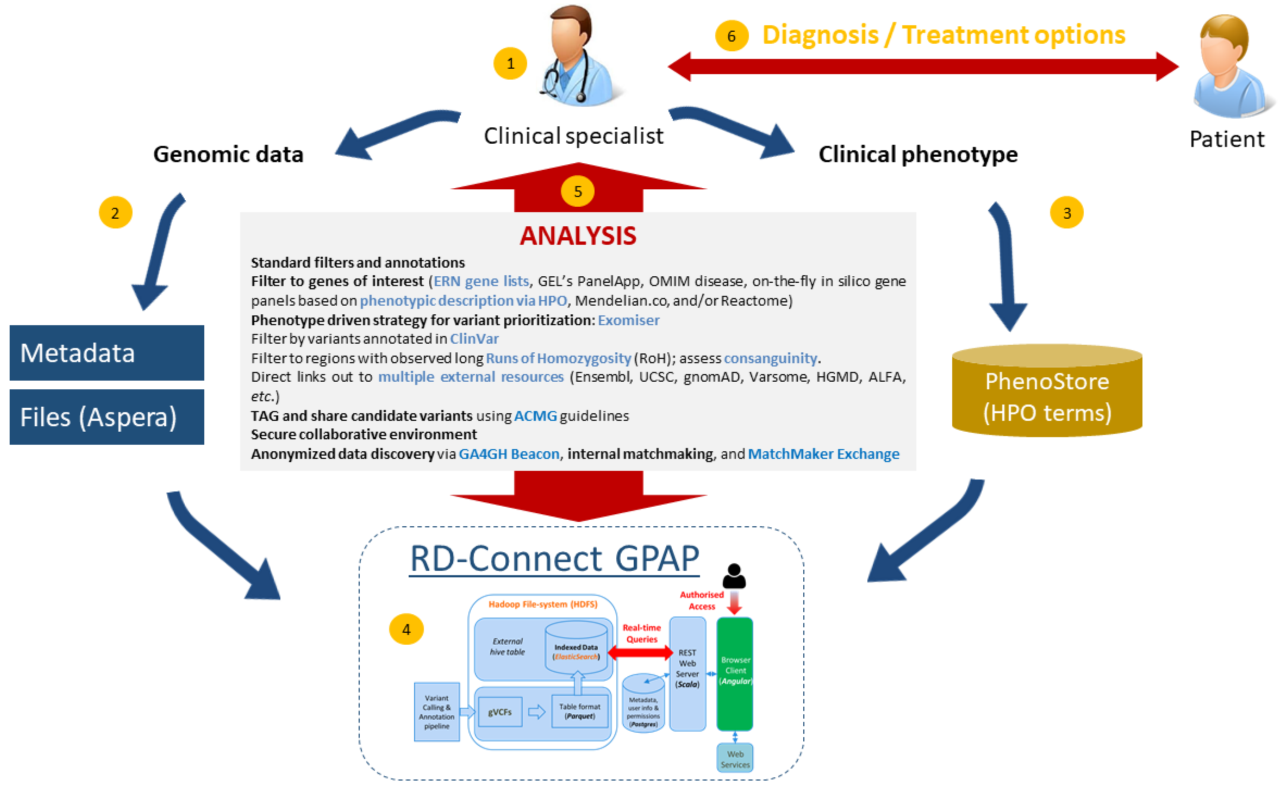

Supplementary Table 1. Pre-screening performed before WES.

Disease group	Genetic pre-screening	Lab pre-screening	Clinical and other pre-screening
Intellectual disability with epilepsy	Karyotype; CGH array; *SCN1A*; Angelman; *CLP1* founder (Turkey)	Acylcarnitines, VLCFA, AFP, Urinary organic acid, amino acids	Brain MRI, EEG
Intellectual disability without epilepsy	Karyotype; CGH array; FRAXA (fragile X); *CLP1* founder (Turkey)	Acylcarnitines, VLCFA, AFP, Urinary organic acid, amino acids	Brain MRI
Epilepsy	*SCN1A*	Acylcarnitines, VLCFA, AFP, Urinary organic & amino acids	Brain MRI, EEG
Brain malformations	Karyotype	CK	Brain MRI, EEG
Leukodystrophies	Direct gene testing if biochemical test reveals specific enzyme deficiency	VLCA; phytanic acid, pristanic acid; lysosomal enzymes, hexosaminidase	Brain MRI, EMG; Nerve conduction Velocities
Spinocerebellar ataxias in children	Friedreich ataxia; *CWF19L1* founder (Turkish), *GRM* founder (Roma)	Acylcarnitines, VLCFA, AFP, Urinary organic acid, amino acids, immun-elpho	Brain MRI, Nerve conduction velocities, EMG, ophthalmology
Spastic paraplegia in children	*SPG4/SPG7*	Acylcarnitines, VLCFA, AFP, Urinary organic acid, amino acids, serum immuno electrophoresis	Brain MRI, spine MRI, nerve conduction velocities, EMG, ophthalmology
CMT and hereditary neuropathies	*PMP22* del/dup; *SMN1* deletion; *NTRK1* Turkish founder, HMSN Lom/Russe/CMT4C gypsy founder; *CLP1* founder (Turkey)	Acylcarnitines, VLCFA, AFP, Urinary organic acid, amino acids	Nerve conduction velocities, EMG, Repetitive stimulation
Muscular dystrophies and myopathies (congenital or childhood-onset)	Dystrophin MLPA; Myotonic Dystrophy type 1; *SMN1* deletion; FSHD type 1; *POMT1* founder (Turkey), *ETFDH* founder (Turkey), *PLEC* founder (Turkey)	CK, Pompe dried blood spot	Muscle biopsy (histology), Muscle biopsy (immunohistochemistry, immunoblotting), Brain MRI, Nerve conduction velocities, EMG, Repetitive stimulation, echocardiography, ECG
Congenital myasthenic syndromes	*CHRNE* sequencing; *CHAT* founder (Turkey)	CK	Nerve conduction velocities, EMG, Repetitive stimulation, Muscle biopsy
Mitochondrial disorders that primarily affect the nervous system and muscle	MELAS, MERRF, NARP/MILS mtDNA mutations, *ETFDH* founder (Turkey), mtDNA single deletion (in blood)	Lactate, ALT/AST/GGT, glucose, blood cell count, CK	Muscle biopsy (COX, SDH), Brain MRI, Nerve conduction velocities, EMG, ophthalmology, echocardiography, ECG, EEG, audiology
Cerebellar hypoplasia or atrophy	*CLP1* founder (Turkey), CGH array (if combined with ID)		Brain MRI, Nerve conduction velocities, EMG, ophthalmology

Abbreviations: CGH: comparative genomic hybridization; VLCFA: very long chain fatty acids; AFP: alpha fetoptotein; CK: creatine kinase; ALT: alanine aminotransferase;/AST: aspartate transaminase; /GGT: gamma glutamyl transferase; MRI: magnetic resonance imaging; EEG: electro-encephalography, ECG, electrocardiography; EMG: emelctromyography; COX: cytochrome c oxidase; SDH: succinate dehydrogenase

Supplementary Table 2. Variants identified in the different disease groups.

Novel genes: not listed as disease genes in OMIM until 31/12/2020.

A. Intellectual Disabilities					
**Family ID**	**Sex**	**Gene**	**Zygosity**	**Variant**	**Reported/Novel**
**Pathogenic variants in known genes (n=11 families, 13 cases)**					
FMAL005_01	F	***RSRC1***	homozygous	c.538G>C, p.Ala180Pro	novel
FDIY0038_01 +1	M	***MAN1B1***	homozygous	c.1000C>T, p.Arg334Cys	reported
FDIY042_01	M	***KIF1C***	homozygous	c.2005C>T, p.Arg669Ter	novel
FIZM018_01 +1	M	***MED12***	hemizygous, XLR	c.5898dupC, p.Ser1967GlnfsTer84	reported
FMAL013_01	M	***MED12***	hemizygous, XLR	c.4115A>G, p.Asn1372Ser	novel
FIZM042_01	M	***PTCHD1***	hemizygous, XLR	c. 2535A>G, p.Ile845Met	reported
FIZM043_01	M	***AMMECR1***	hemizygous, XLR	c.973C>A, p.Pro325Thr	novel
FIZM053_01	M	***SQSTM1***	homozygous	c.311_312del,p.Glu104ValfsTer48	reported
FIZM055_04	M	***ASH1L***	homozygous	c.7756G>A, p.Gly2591Ser	novel
FMAL010_01	F	***PIEZO2***	homozygous	c.4501T>G, p.Leu1501Val	novel
FMAL041_01	M	***ERCC6***	homozygous	c.1992+3	novel
**Novel candidates (n=2 families, 4 cases)**					
FDIY0034_01 +2	M	***USP38***	double homozygous	c.2257C>G, p.Gln753Glu<br>c.2489A>T, p.Asp830Val	novel gene
FIZM030_01	F	***HIST1H4C***	heterozygous, de novo	c.275A>G, p.Lys92Arg	novel gene
**Unsolved (n = 7 families, 9 cases)**					

B. Brain malformations					
**Family ID**	**Sex**	**Gene**	**Zygosity**	**Variant**	**Reported**
**Pathogenic variants in known genes (n=21 families, 28 cases)**					
FMAL011_01 double trouble	F	***OCLN***<br><br>***MCCC2***	homozygous<br><br>homozygous	c.173_194del, p.Trp58PhefsTer10<br>c.1015G>A, p.Val339Met	reported<br><br>reported
FDIY018_01	F	***TCF20***	heterozygous, de novo	c.2381A>C, p.Gln794Pro	novel
FDIY001_01 + 5	M	***VPS13B***	compound heterozygous	c.412+1G>T;<br>c.7504+40A>T	reported<br>novel
FDIY023_01 + 1	M	***TAF1***	hemizygous, XLR	c.4603A>G, p.Ile1535Val	novel
FDIY025_01	F	***SCARB2***	homozygous	c.277G>A, p.Glu93Lys	reported
FDIY030_01	F	***TTC1***	homozygous	c.784T>G, p.Phe262Val	reported
FIZM063_01 +1	F	***TTC1***	homozygous	c.784T>G, p.Phe262Val	reported
FIZM002_01	F	***PORCN***	het de novo, XLD	c.283C>T, p.Arg95Ter	reported
FIZM010_01	F	***TLK2***	homozygous	c.163A>G, p.Lys55Glu	self-reported
FIZM014_01	F	***TBCD***	homozygous	c.898C>T, p.Leu300Phe	novel
FIZM015_01	F	***CASK***	het de novo, XLD	c.1910G>A, p.Gly637Asp	reported
FIZM021_01	M	***GALK1***<br>***ARX***	homozygous<br>hemizygous, XLR	c.110G>A, p.Arg37His<br>c.610C>A, p.Arg204Ser	novel<br>novel
FIZM024_01	F	***LAMB1***	homozygous	c.4238G>A, p.Gly1413Glu	novel
FIZM028_01	F	***CC2D1A***	homozygous	c.2027G>T, p.Gly676Val	reported
FIZM055_01	M	***COL18A1***	homozygous	c.1567_1568delCT, p.Leu523ValfsTer48	reported
FIZM056_01	M	***CNPY3***	homozygous	c.79G>T, p.Ala27Ser	novel
FMAL007_01	M	***CLP1***	homozygous	c.419G>A, p.Arg140His	reported
FMAL038_01	F	***CLP1***	homozygous	c.419G>A, p.Arg140His	reported
FMAL016_01	F	***KATNB1***	homozygous	c.100C>T, p.Arg34Trp	novel
FMAL061_01	M	***STIL***	homozygous	c.3637G>T, p.Glu1214Ter	novel
FMAL088_01	F	***RTTN***	homozygous	c.6154-16_6154-13delCTCT	novel splice variant
**Novel candidates (n=6 families, 10 cases)**					
FMAL006_01	F	***WDR91***	homozygous	c.1395+1G>A	novel gene
FDIY021_01 + 1	M	***SPP1***	homozygous	c.120C>T(p.=)<br>p.Glu105Ter	novel gene
FDIY028_01 + 3	M	***DHX37***	homozygous	c.661G>C,p.Ala221Pro	novel gene
FIZM011_01	M	***PTPMT1***	homozygous	c.255G>T, p.Gln85His	novel gene
FMAL035_01	M	***NAA60***	homozygous	c.130C>T; p.Arg44Cys	novel gene
FDIY029_01	M	***UFSP2***	homozygous	c.344T>A; p.Val115Glu	novel gene
**Unsolved (n = 1 family, 1 case)**					

**C. Leukodystrophies**					
**Family ID**	**Sex**	**Gene**	**Zygosity**	**Variant**	**Reported**
**Pathogenic variants in known genes (n=12 families, 15 cases)**					
FDIY024_01 + 1	M	***COL4A1***	homozygous	c.3832G>A, p.Gly1278Ser	self-reported
FIZM062_01	F	***RNASET2***	homozygous	c.194A>G, p.His65Arg	novel
FMAL033_01	M	***RNASET2***	homozygous	c.550T>C, p.Cys184Arg	reported
FMAL001_01	M	***AASS***	homozygous	c.565A>G, p.Arg189Gly	novel
FMAL002_01	M	***TACO1***	homozygous	c.472dupC, p.His158ProfsTer8	reported
FMAL017_01	M	***SAMHD1***	homozygous	c.490C>T, p.Arg164Ter	reported
FMAL019_01	M	***SAMHD1***	homozygous	c.490C>T, p.Arg164Ter	reported
FMAL021_01	M	***ACER3***	homozygous	c234G>A, p.Trp78Ter	novel
FMAL031_01	F	***STAMBP***	homozygous	c.188A>G, p.Tyr63Cys	novel
FMAL067_01	F	***HMBS***	homozygous	c.500G>A, p.Arg167Gln	reported
FMAL023_01 + 1	F	***TUBGCP2***	homozygous	c.931G>A, p.Glu311Lys	self-reported
FMAL064_01	F	***CRB1***	homozygous	deletion	novel CNV
FMAL064_04	M	***PLA2G6***	homozygous	deletion	novel CNV
**Novel candidates (n= 3 family, 3 case)**					
FMAL057_01	F	***DDB1***	homozygous	c.2566+4A>G	novel gene
FMAL059_01	M	***SPATA5L1***	homozygous	c.85T>G; p.Cys29Gly	novel gene
FMAL037_01	M	***ZNF92***	homozygous	deletion	novel gene
**Unsolved (n = 1 family, 1 case)**					

D. ID with epilepsy					
**Family ID**	**Sex**	**Gene**	**Zygosity**	**Variant**	**Reported**
**Pathogenic variants in known genes (n= 38 families, 45 cases)**					
FIZM019_01	F	***PNPO***	homozygous	c.674G>A, p.Arg225His	reported
FIZM022_01	M	***ADSL***	homozygous	c.268G>A, p.Ala90Thr	reported
FDIY017_01+1	M	***ADSL***	homozygous	c.1288G>A, p.Ala2354Ser	novel
FMAL058_01	F	***ADSL***	homozygous	c.268G>A, p.Ala90Thr	novel
FIZM038_01	F	***ATP6V0A2***	homozygous	c.745G>A, p.Val249Leu	reported
FMAL060_01 +1	F	***MECP2***	het de novo, XLD	c.842delG,p.Gly281AlafsTer20	reported
FDIY007_01	M	***TPP1***	homozygous	c.638C>T, p.Ser196Leu	novel
FDIY011_01	F	***CDKL5***	het de novo, XLD	c.587C>T, p.Ser196Leu	reported
FDIY0037_01 +1	F	***CDKL5***	het de novo, XLD	c.401G>A, p.Arg134Gln	novel
FDIY035_01	F	***OXR1***	homozygous	c.1762A>T, p.Lys588Ter	novel
FDIY014_01	M	***GAMT***	homozygous	c.144dupC, p.Tyr49LeufsTer36	novel
FDIY016_01	F	***ASXL3***	heterozygous, de novo	c.6072delT, p.Pro2028TrpfsTer51	novel
FMAL012_01	F	***ASXL3***	heterozygous, de novo	c.6072delT, p.Pro2028TrpfsTer51	novel
FDIY027_01	M	***MFSD8***	homozygous	exon 2 deletion	novel CNV
FDIY0039_01	M	***WWOX***	homozygous	c.716T>G, p.Leu239Arg	reported
FIZM006_01	M	***WWOX***	homozygous	c.716T>G, p.Leu239Arg	reported
FIZM029_01	M	***WWOX***	homozygous	c.689A>C, p.Gln230Pro	reported
FMAL028_01	M	***WWOX***	homozygous	c.716T>G, p.Leu239Arg	reported
FDIY040_01	F	***MAP2K1***	heterozygous de novo	c.199G>A, p.Asp67Asn	reported
FIZM004_01	F	***POLR1A***	homozygous	c.4498C>T, p.Arg1500Cys	novel
FIZM016_01	M	***ARX***	hemizygous, XLR	c.1448+1G>A	novel
FIZM040_01	M	***RAB3GAP2***	homozygous	c.1201_1202delGTinsTA, p.Val401Ter	novel
FIZM046_01	M	***CUX2***	heterozygous, de novo	c.3758delA, p.His1253ProfsTer20	novel
FIZM049_01	F	***PLA2G6***	homozygous	c.1912G>A, p.Gly638arg	novel
FIZM052_01 + 1	M	***ALG3***	homozygous	c. 100C>T, p.Hist34Tyr	novel
FMAL020_01+ 1	M	***CNTNAP2***	homozygous	c.847C>T; p.Arg283Cys	reported
FMAL029_01	M	***DENND5A***	homozygous	c.1964T>G, p.Leu655Ter	novel
FMAL048_01	M	***KMT2C***	homozygous	c.13174C T, p.Pro4392Ser	novel
FMAL062_01	M	***SYNGAP1***<br><br>***UFSP2***	heterozygous, de novo<br>homozygous	c.3963_3964delAG, p.Ala1322ProfsTer40<br>c.344T>A; p.Val115Glu	novel<br><br>novel gene
FMAL066_01	F	***SYNGAP1***	heterozygous, de novo	c.3962dupC, p.Ala1322SerfsTer41	novel<br>novel gene
FMAL070_01	M	***AP3B2***	homozygous	c.2921_2922delCT,p.Pro974ArgfsTer5	reported
FMAL072_01	F	***PCDH12***	homozygous	c.451C>T, p.Arg151Ter	novel
FMAL096_01 +1	M	***PCDH12***	homozygous	c.451C>T, p.Arg151Ter	novel
FMAL074_01	M	***SCN2A***	homozygous	c.1976G>A, p.Gly659Asp	reported
FMAL084_01	M	***SCN2A***	heterozygous, de novo	c.1177-2A>C	novel
FMAL076_01 +1	M	***PIGT***	homozygous	c.644A>G, p.Tyr215Cys	novel
FMAL098_01	M	***NECAP1***	homozygous	c.370delC, p.Gln124ArgfsTer	novel
FIZM035_01	M	***PTPN23***	homozygous	c.4597_4598delAGinsTT, p.Ser1533Phe	novel

**Novel candidates (n=13 families, 15 cases)**					
FDIY026_01	M	***KATNAL2***	homozygous	c.1174T>C, p.Ser392Pro	novel gene
FIZM031_01	M	***GFRA1***	homozygous	c.611C>T, p.Pro204Leu	novel gene
FDIY0033_01	M	***CCDC82***	homozygous	c.1036delG, p.Ala346LeufsTer3	novel gene
FIZM034_01	F	***DHX37***	homozygous	c.1105G>A, p.Val369Met	novel gene
FIZM045_01	F	***XAB2***	homozygous	c.2047C>T, p.Arg683Cys	novel gene
FIZM044_01	M	***MAN1A2***	homozygous	c.553A>T, p.Lys185Ter	novel gene
FMAL077_01	M	***EEF1D***	homozygous	c.947G>A, p.Trp316Ter	novel gene
FMAL030_01	F	***UBAP2***	homozygous	c.970A>G, p.Ile324Val	novel gene
FMAL078_01	M	***TFAP2E***	homozygous	c.671G>A, p.Arg224Gln	novel gene
FMAL079_01	M	***KNDC1***	homozygous	c.3560T>G, p.Leu1187Trp	novel gene
FIZM050_01 +1	M	***KRBOX4***	hemizygous, XLR	c.142G>T, p.Gly48Trp	novel gene
FMAL051	M	**Chr9 del**	homozygous	AK3/CDC37L1/CDC37L1-DT/PLPP6/SPATA6L deletion	novel CNV
FMAL086_01+1	M	**ACSM5del**	homozygous	Chr16 deletion	novel CNV
**Unsolved (n = 7 families, 8 cases)**					

E. Neuromuscular diseases						
**Family ID**	**Sex**	**Subgroup**	**Gene**	**Zygosity**	**Variant**	**Reported**
**Pathogenic variants in known genes (n= 37 families, 51 patients)**						
FDIY015_01 + 1	F	Mitoch	***FOXRED1***	homozygous	c.473G>T, p.Gly158Val	novel
FDIY022_01	M	Mitoch	***DLAT***	homozygous	c.1728C>G, p.Phe576Leu	novel
FDIY0031_01	F	CMT	***CCDC28B***	homozygous	c.685C>T, p.Pro229Ser	novel
FDIY0032_01 +2	M	Mitoch	***FOLR1***	homozygous	c.610C>T, p.Arg204Ter	reported
FDIY041_01 + 1	F	Mitoch	***FOLR1***	homozygous	c.493+2T>C	reported
FIZM009_01	F	CMD	***COL6A2***	homozygous	c.1332+242_1332+243insCA	novel
FIZM012_01 +1	M	Mitoch	***NDUFA12***	homozygous	c.121dupG,p.Glu41GlyfsTer10	reported
FIZM027_01 +1	F	Myopathy	***CACNA1S***	homozygous	c.2366G>A, p.Arg789His	self-reported
FIZM037_01	M	Mitoch	***ETFDH***	homozygous	c.1130T>C, p.Leu377Pro	reported
FMAL065_01	M	Myopathy	***ETFDH***	homozygous	c.1130T>C, p.Leu377Pro	reported
FIZM054_01	M	Mitoch	***TUFM***	homozygous	c.1016G>A, p.Arg339Gln	novel
FMAL014_01	M	Myopathy	***COL12A1***	heterozygous de novo	c.5765G>A, p.Gly1922Glu	novel
FIZM007_01	M	CMS	***DPAGT1***	homozygous	c.339T>G, p.Phe113Leu	novel
FIZM020_01	F	Mitoch	***RMND1***	homozygous	c.158T>A, p.Val264Glu	novel
FIZM041_01	M	Mitoch	***COQ4***	homozygous	c.437T>G, p.Phe146Cys	reported
FIZM057_01	M	CMT	***USH2A***	homozygous	c.7047G>A, p.Trp2349Ter	novel
FIZM058_01 +1	M	CMS	***COLQ***	homozygous	c.414G>A, p.Trp138Ter	reported
FMAL024_01	M	CMS	***COLQ***	homozygous	c.414G>A, p.Trp138Ter	reported
FMAL087_01	M	CMS	***COLQ***	homozygous	c.1169A>G, p.Asn381Ser	novel
FIZM061_01	F	Myopathy	***CCDC78***	compound heterozygous	c.1272_1274dupGAG,p.Arg424dup; c.287G>A, pArg96Gln	novel<br>novel
FMAL003_01	F	Myopathy	***ASXL3***	compound heterozygous	c.6078_6081delTCCA, p.Pro2028TrpfsTer51	novel
FMAL015_05	F	CMD	***POMT1***	compound heterozygous	c.598G>C, p.Ala200Pro; p.Asn89GlnfsTer8	reported
FMAL025_01	F	CMT	***MMAA***	homozygous	c.1117G>T, p.Glu373Ter	novel
FMAL027_01	F	Myopathy	***ISPD***	homozygous	c.1026+6T>A	novel
FMAL039_01	M	CMT	***SLC39A8***	homozygous	c.545T>C, p.Ile182Thr	novel
FMAL043_01	M	CMS	***CHRND***	homozygous	c.697C>T, p.Arg233Cys	reported
FMAL044_01 +1	F	CMT	***SH3TC2***	compound heterozygous	c.1897delG, p.Ala633ProfsTer12<br>c.1747_48del,p.Arg583AlafsTer4	novel<br><br>reported
FMAL097_01+2	F	CMT	***SH3TC2***	homozygous	c.1552delG, p.Ala518ProfsTer6	novel
FMAL045_01	F	CMT	***BRAT1***	compound heterozygous	c.1507C>T,p.Pro503Ser;<br>c.1376G>A, p.Ser459Asn	reported<br>reported
FMAL047_01	F	CMT	***GDAP1***	homozygous	c.786delG, p.Phe263LeufsTer22	reported
FMAL099_01 +1	M	CMT	***GDAP1***	homozygous	c.689C>A, p.Thr230Asn	novel
FMAL050_01 +1	F	Myopathy	***CLCN1***	homozygous	c.1063G>C, p.Gly355Arg	novel
FMAL052_01	F	Muscular dystrophy	***SGCA***	homozygous	c.850C>T, p.Arg284Cys	reported
FMAL080_01	F	Vici syndr	***EPG5***	homozygous	c.1252+1G>A	reported
FMAL081_01	F	Muscle weakness	***PEX1***	homozygous	c.1099delC, p.Gln367LysfsTer20	novel
FMAL083_01 +1	F	Ehler-Danlos	***PLOD1***	homozygous	c.975+2_975+3insTT	novel
FMAL094_01	M	CMT	***CNTNAP1***	homozygous	c.2636G>A, p.Arg879Gln	novel
**Novel candidates (n=3 families, 4 patients)**						
FIZM026_01 +1	M	Myopathy	***FBXO34***	homozygous	c.482A>G, p.Lys161Arg	novel gene
FMAL034_01	M	Myopathy	***NPAP1***	homozygous	c.3407C>G, p.Ser1136*	novel gene
FMAL046_01	F	CMT	***ARHGAP19***	homozygous	c.451C>A , p.Glu151Lys	novel gene
**Unsolved (n = 8 families, 10 patients)**						

CMT: Charcot-Marie-Tooth disease; CMD: congenital muscular dystrophy; CMS: congenital myasthenic syndrome.

F. Ataxias and spastic paraparesis						
**Family ID**	**Sex**	**Subgroups**	***Gene***	**Zygosity**	**Variant**	**Reported**
**Pathogenic variants in known genes (n=18 families, 27 cases)**						
FDIY0036_01	F	Ataxia	***GNPTG***<br>***UFSP2***	homozygous<br>homozygous	c.277G>A, p.Glu116Lys<br>c.344T>A; p.Val115Glu	novel<br>novel gene
FMAL036_01+1	M	SPG	***GNPTG***	homozygous	c.367A>G,p.Met123Val	novel
FDIY043_01 +1	M	Ataxia	***GRM1***	homozygous	c.1524T>G,p.Asp508Glu	novel
FIZM033_01	F	Ataxia	***KIAA0586***	homozygous	c.38delA,<br>p.Lys13ArgfsTer6	novel
FIZM051_01	F	Ataxia	***ATCAY***	homozygous	c.552C>G, p.Tyr184Ter	novel
FMAL009_01<br>+3	F	Ataxia	***PRKCG***	homozygous	c.1769T>C,<br>p.Leu590Pro	novel
FMAL026_01<br>+1	F	SPG	***ALS2***	homozygous	c.470G>A, p.Cys157Tyr	reported
FMAL049_01	M	SPG	***ALS2***	homozygous	c.4573dupG,<br>p.Val1525GlyfsTer17	novel
FMAL040_01	F	Spastic<br>ataxia	***SACS***	homozygous	c.2225G>A,<br>p.Arg742Gln	novel
FMAL042_01	F	Spastic<br>ataxia	***SACS***<br><br>***PARK7***	homozygous<br>homozygous	c.2182C>T,<br>p.Arg728Ter<br>deletion of 16 exons	reported<br><br>novel CNV
FMAL053_01<br>+1	M	Ataxia	***ATP8A2***	homozygous	c.1756C>T,<br>p.Arg586Ter	reported
FMAL055_01<br>+1	F	SPG	***SPG11***	homozygous	c.3075dupA,<br>p.Glu1026ArgfsTer4	reported
FMAL071_01	M	Ataxia	***SPTBN2***<br><br>***UFSP2***	homozygous<br><br>homozygous	c.2330dupA,<br>p.His777GlnfsTer106<br>c.542delT	novel<br><br>novel gene
FMAL073_01	F	Ataxia	***CACNB4***	homozygous	c.8C>T, p.Ser3Phe	novel
FMAL082_01	M	Movement<br>disorder	***ADCY5***	heterozygous,<br>de novo	c.2090G>A,<br>p.Gly697Asp	novel
FMAL085_01	M	Ataxia	***ATM***	homozygous	c.3161C>A,<br>p.Pro1054His	reported
FMAL091_01<br>+1	F	Ataxia	***TMEM240***	homozygous	c.47C>A, p.Ser16Ter	novel
FIZM032_01	M	Ataxia	***SNX14***<br>***TMLHE***	homozygous<br>hemizygous<br>XLR	c.2670T>A,p.Cys890Ter<br>deletion	reported<br>novel CNV
**Unsolved n=2 family, 2 case**						

SPG: spastic paraparesis

Supplementary Table 3. **Sample breakdown for the three recruiting centres.**

Centre	Solved	Novel candidate	Unsolved
Diyarbakir (n=35 families)	n=24, 69%	n=6, 17%	n=5, 14%
Izmir (n=58 families)	n=41, 71%	n=8, 14%	n=9, 15%
Malatya (n=97 families)	n=71, 74%	n=13, 13%	n=13, 13%

Supplementary Table 4. Main clinical groups and number of patients/ families.

Disease category	Number of patients (families)	Solved Fam/Pat	Novel candidate Fam/Pat	Unsolved Fam/Pat
Brain malformations	28 families / 39 cases	21 / 28<br>75%	6 / 10<br>21%	1 / 1<br>4%
Leukodystrophies	16 families / 19 cases	12 / 15<br>75%	3 / 3<br>19%	1 / 1<br>6%
Intellectual disability (ID) with epilepsy	58 families / 68 cases	38/ 45<br>66%	13 / 15<br>22%	7 / 8<br>12%
Intellectual disability (ID)	20 families / 26 cases	11 / 13<br>55%	2 / 4<br>10%	7 / 9<br>35%
Neuromuscular diseases	48 families / 65 cases	37 / 51<br>77%	3 / 4<br>6%	9 / 10<br>17%
Ataxias and spastic paraparesis	20 families / 29 cases	18 / 27<br>90%	0 / 0<br>0%	2 / 2<br>10%
**TOTAL**	**190 families / 246 cases**	**137/179<br>72%**	**27/36<br>14%**	**26/31<br>14%**

Supplementary Table 5. Main clinical groups and solved rates in known disease genes in WES compared to next generation panel testing approach.

Experience paediatric neurologists defined the clinical categories. Due to the overlapping phenotypes over half of the patients with genetic diagnosis in a known disease gene would have been missed on common gene panel testing. We used the following panels from GeneDx (https://www.genedx.com/test-catalog/testing-directory/#!/by-test/numon on 29/04/2021): Cortical brain malformations panel; Leukodystrophies extended panel; Autism / Intellectual disability panel; Infantile epilepsy panel; Childhood-onset epilepsy panel; Neuromuscular diseases panel; Congenital myopathy and muscular dystrophy panel; Mitochondrial focussed nuclear gene panel; Charcot-Marie-Tooth disease panel; Congenital myasthenic syndromes panel; Spinocerebellar ataxia and related disorders panel; Comprehensive spastic paraplegia panel.

Disease category	Number of families with known gene defect	Number of families with gene not on NGS panel
Brain malformations	21 families	18 families (86%)
Leukodystrophies	12 families	6 families (50%)
ID with epilepsy	38 families	18 families (47%)
Intellectual disability (ID)	11 families	8 families (73%)
Neuromuscular diseases	37 families	18 families (48%)
Ataxias and spastic paraparesis	18 families	8 families (44%)
**TOTAL**	**137 families**	**71 families (52%)**

Supplementary Dataset: ACMG classification of identified variants in known disease genes. Grey highlighted are homozygous variants in genes previously only associated with autosomal dominant or de novo inheritance.

Refernces

1. Celestino-Soper PB, Shaw CA, Sanders SJ, et al. Use of array CGH to detect exonic copy number variants throughout the genome in autism families detects a novel deletion in TMLHE. *Hum Mol Genet*. Nov 15 2011;20(22):4360-70. doi:10.1093/hmg/ddr363
2. Celestino-Soper PB, Violante S, Crawford EL, et al. A common X-linked inborn error of carnitine biosynthesis may be a risk factor for nondysmorphic autism. *Proc Natl Acad Sci U S A*. May 22 2012;109(21):7974-81. doi:10.1073/pnas.1120210109
3. Grunert SC, Stucki M, Morscher RJ, et al. 3-methylcrotonyl-CoA carboxylase deficiency: clinical, biochemical, enzymatic and molecular studies in 88 individuals. *Orphanet J Rare Dis*. May 29 2012;7:31. doi:10.1186/1750-1172-7-31
